# New physiological insights using multi-TE ASL MRI measuring blood–brain barrier water exchange after caffeine intake

**DOI:** 10.1007/s10334-024-01219-x

**Published:** 2025-01-25

**Authors:** Amnah Mahroo, Mareike Alicja Buck, Simon Konstandin, Jörn Huber, Daniel Christopher Hoinkiss, Jochen Hirsch, Matthias Günther

**Affiliations:** 1 Imaging Physics, Fraunhofer Institute for Digital Medicine MEVIS, Max-von-Laue-Straße 2, 28359 Bremen, Germany; 2 MR-Imaging and Spectroscopy, University of Bremen, Bremen, Germany; 3 Mediri GmbH, Heidelberg, Germany

**Keywords:** Blood–brain Barrier, Permeability, Magnetic Resonance Imaging, Arterial Spin Labeling, Caffeine

## Abstract

**Objectives:**

Caffeine, a known neurostimulant and adenosine antagonist, affects brain physiology by decreasing cerebral blood flow. It interacts with adenosine receptors to induce vasoconstriction, potentially disrupting brain homeostasis. However, the impact of caffeine on blood–brain barrier (BBB) permeability to water remains underexplored. This study investigated the water exchange via the BBB in a perturbed physiological condition caused by caffeine ingestion, using the multiple echo time (multi-TE) arterial spin labeling (ASL) technique.

**Material and methods:**

Ten healthy, regular coffee drinkers (age = 31 ± 9 years, 3 females) were scanned to acquire five measurements before and six measurements after caffeine ingestion. Data were analyzed with a multi-TE two-compartment model to estimate exchange time (Tex), serving as a proxy for BBB permeability to water. Additionally, cerebral blood flow (CBF), arterial transit time (ATT), and intravoxel transit time (ITT) were investigated.

**Results:**

Following caffeine intake, mean gray matter CBF showed a significant time-dependent decrease (*P* < 0.01). In contrast, Tex, ATT, and ITT did not exhibit significant time-dependent change. However, a non-significant decreasing trend was observed for Tex and ITT, respectively, while ATT showed an increasing trend over time.

**Discussion:**

The observed decreasing trend in Tex after caffeine ingestion suggests a potential increase in water flux across the BBB, which may represent a compensatory mechanism to maintain brain homeostasis in response to the caffeine-induced reduction in CBF. Further studies with larger sample sizes are needed to validate and expand upon these findings.

## Introduction

Caffeine (1,3,7-trimethylxanthine) is one of the most widely consumed psychostimulant [1] compounds and is known to modulate brain physiology [2]. It acts as an antagonist to adenosine, an inhibitory neurotransmitter, by binding to adenosine receptors and, as a result, improves alertness and vigilance [3]. Adenosine has been reported to play a critical role in neural transmission and in regulating cerebral blood flow (CBF) [4, 5].

By binding with adenosine receptors, A_2A_ and A_2B_, caffeine produces a vasoconstrictive effect on smooth muscles [6]. Various studies have demonstrated modulated neural activity and decreased CBF in the brain in response to caffeine ingestion [7–9]. Moreover, by blocking adenosine receptors, caffeine increases peripheral vascular resistance which leads to increased blood pressure, causing a reduction in blood flow velocity. Besides all these physiological changes, the brain—being highly adaptive and resilient—possesses mechanisms to maintain and protect homeostasis within the tissue [10]. This provides an opportunity to investigate how different brain structures, for example, blood–brain barrier (BBB), respond to such physiological changes in a healthy human.

BBB is a neuroprotective layering of various cells around brain vessels that tightly regulate the movement of substances in the brain and maintain brain homeostasis [11]. Previous studies reported that the adenosine receptor signaling modulates BBB permeability [12, 13]. However, the impact of caffeine on the BBB permeability to water and its interplay with decreased perfusion has not been widely studied. A few studies reported that the chronic use of caffeine may play a neuroprotective role in animal models of Alzheimer’s disease and Parkinson’s disease [14, 15]. A recent study applied a non-invasive MRI method of water-extraction-with-phase-contrast-arterial-spin-tagging (WEPCAST) and reported that the BBB permeability remains unchanged in response to caffeine challenge [16]. The authors further reported that the water extraction fraction significantly increased while the brain perfusion decreased in a time dependent manner and the resulting BBB permeability surface area product remained constant.

In this study, we aim to investigate the influence of caffeine ingestion on water exchange via the BBB in healthy humans. We applied an emerging, non-invasive MRI method of multiple echo time (multi-TE) arterial spin labeling (ASL) to measure the exchange time of the labeled water transitioning from the capillaries into the tissue as a proxy measure of BBB permeability. Additionally, simulations were conducted to examine parameter interdependence using one-stage and two-stage model fitting approaches.

## Methods

Multi-TE ASL data offers the ability to distinguish blood and tissue compartments in the ASL signal based on transverse relaxation (T2), which differs significantly at 3 T. Utilizing this technique, an extended multi-TE two-compartment model was recently introduced [17]. This model separates exchange time (Tex)—the time taken by labeled water to move from blood into tissue—and intra-voxel transit time (ITT)—the time required for labeled water to traverse smaller vessels, such as arterioles, before reaching the site of capillary exchange. This addresses the limitation of assuming the instantaneous arrival of labeled water at the exchange site.

The extended model assumes that during the ITT, limited or no exchange occurs, and the signal decays with the T1 of blood only, resulting in a purely blood-based signal component. After ITT, when labeled blood reaches single-cell capillaries, exchange occurs between blood and tissue. The total ASL signal is modeled as a combination of all three components. Detailed model equations can be found in Mahroo et al. (2021).

### Simulations

Accurately estimating CBF and BBB permeability is crucial for understanding brain physiology and pathology. Simulations were conducted to evaluate the impact of one-stage and two-stage model fitting approaches for estimating physiological parameters, including CBF, arterial transit time (ATT), Tex, and ITT. By comparing these approaches, we aim to identify the approach that provides greater accuracy and reliability in parameter estimation, offering insights into the suitability and robustness of these approaches for BBB imaging.

Groundtruth data with a matrix size of 100×100x3 were simulated using the extended multi-TE two-compartment model [17] for two different protocols using MATLAB (MathWorks, Natick, US). Two datasets of multi-TI, single-TE ASL were generated with a sub-bolus duration (SBD) of 450 ms, post-labeling delays (PLD) of 600 ms and 800 ms, and TE of 13.2 ms, resulting in two sets with seven TIs each ranging from 1000 ms to 3400 ms and, 1200 ms to 3600 ms with an increment of 400 ms. A multi-TI, multi-TE ASL dataset was generated with SBD = 1050 ms, PLD = 500 ms, TIs = [1500, 2500, 3500] ms, and eight TEs ranging from 13.8 ms to 207 ms, with an increment of 27.6 ms. Other parameters included 500 ms < ATT < 2500 ms, 0 ms < Tex < 1000 ms, ITT = 200 ms, CBF = 60 ml/100 g/min. Fixed values from the literature [18] were taken for T1 blood = 1664 ms, T1 tissue = 1331 ms, T2 blood = 165 ms and T2 tissue = 85 ms.

**Fig. 1:**
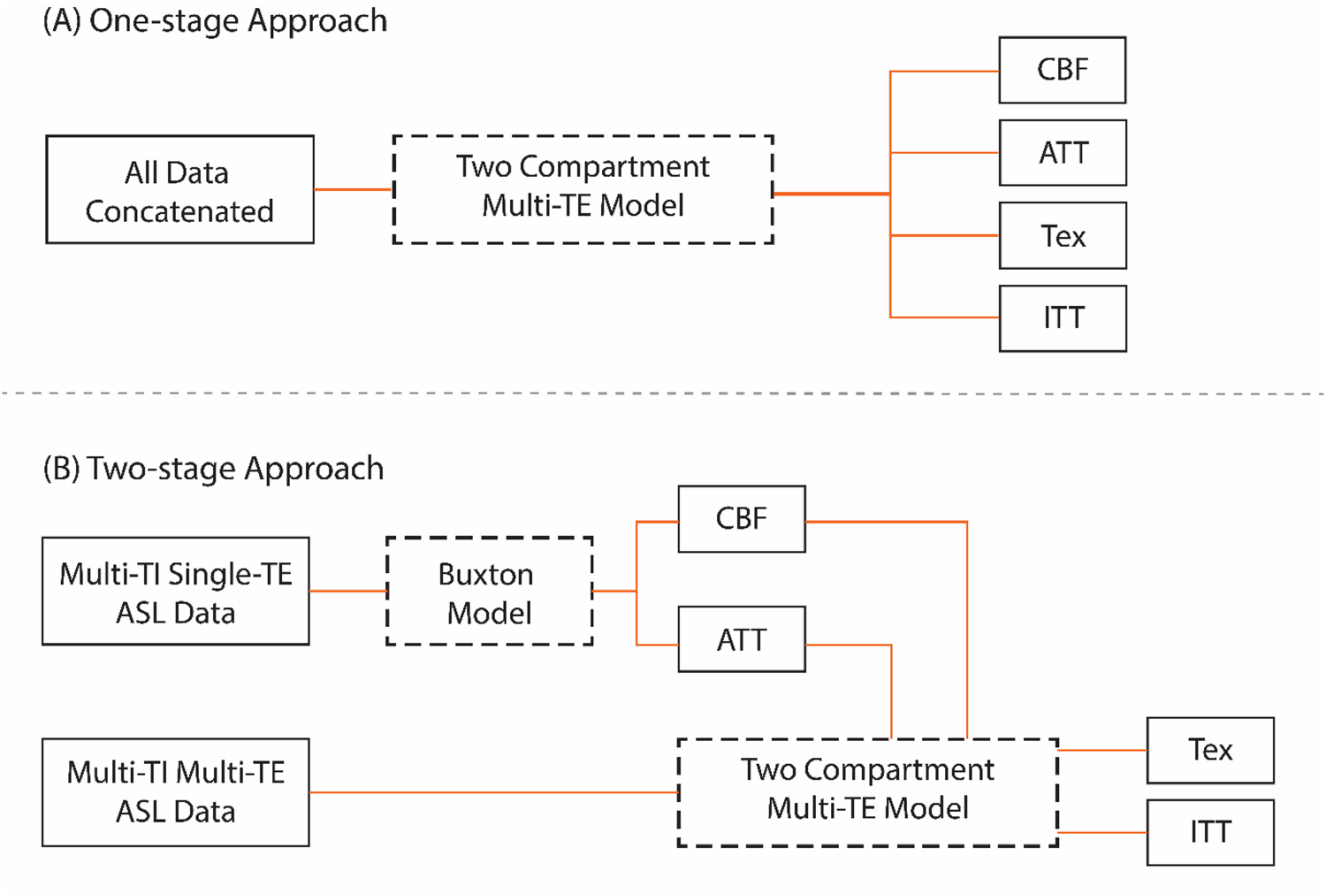
Schematic representation of the one-stage and two-stage model fitting approaches used for parameter estimation. **A** In the one-stage approach, all ASL data (multi-TI and multi-TE) is concatenated and processed simultaneously using the extended two-compartment multi-TE model to estimate CBF, ATT, Tex, and ITT. **B** In the two-stage approach, CBF and ATT are first estimated from multi-TI, single-TE ASL data using the Buxton model. These estimates are then fixed as inputs in the second stage, where the multi-TI, multi-TE ASL data is used along with the extended two-compartment multi-TE model to estimate Tex and ITT

For the one-stage approach, both datasets were concatenated, and all four parameters (CBF, ATT, Tex, and ITT) were estimated using the extended multi-TE two-compartment model. In the two-stage approach, CBF and ATT were first estimated using the multi-TI, single-TE data with the Buxton model [19]. These estimated values were then applied in the second stage with the multi-TI, multi-TE data to estimate Tex and ITT using the extended multi-TE two-compartment model. The workflow of the two fitting approaches is shown in Fig. 1. Model fitting was conducted using the Bayesian non-linear fitting framework of fabber [20] module in Oxford Centre for Functional MRI of the Brain (FMRIB)’s Software Library (FSL) [21].

**Fig. 2:**
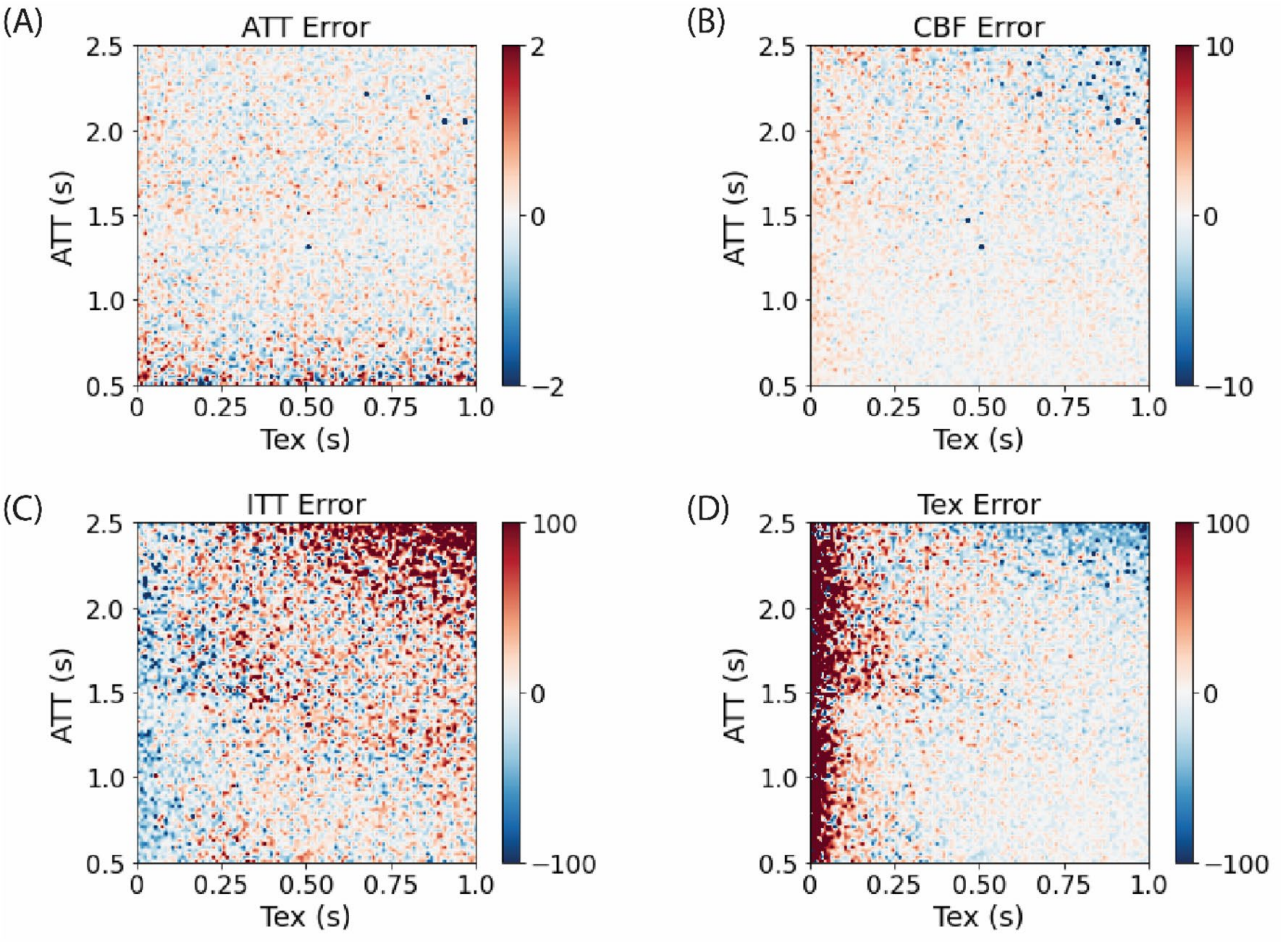
Error maps for estimated parameters across a range of simulated ATT and Tex values using the one-stage approach. Each panel displays the error in a specific parameter: **A** ATT error, **B** CBF error, **C** ITT error, and **D** Tex error

**Fig. 3:**
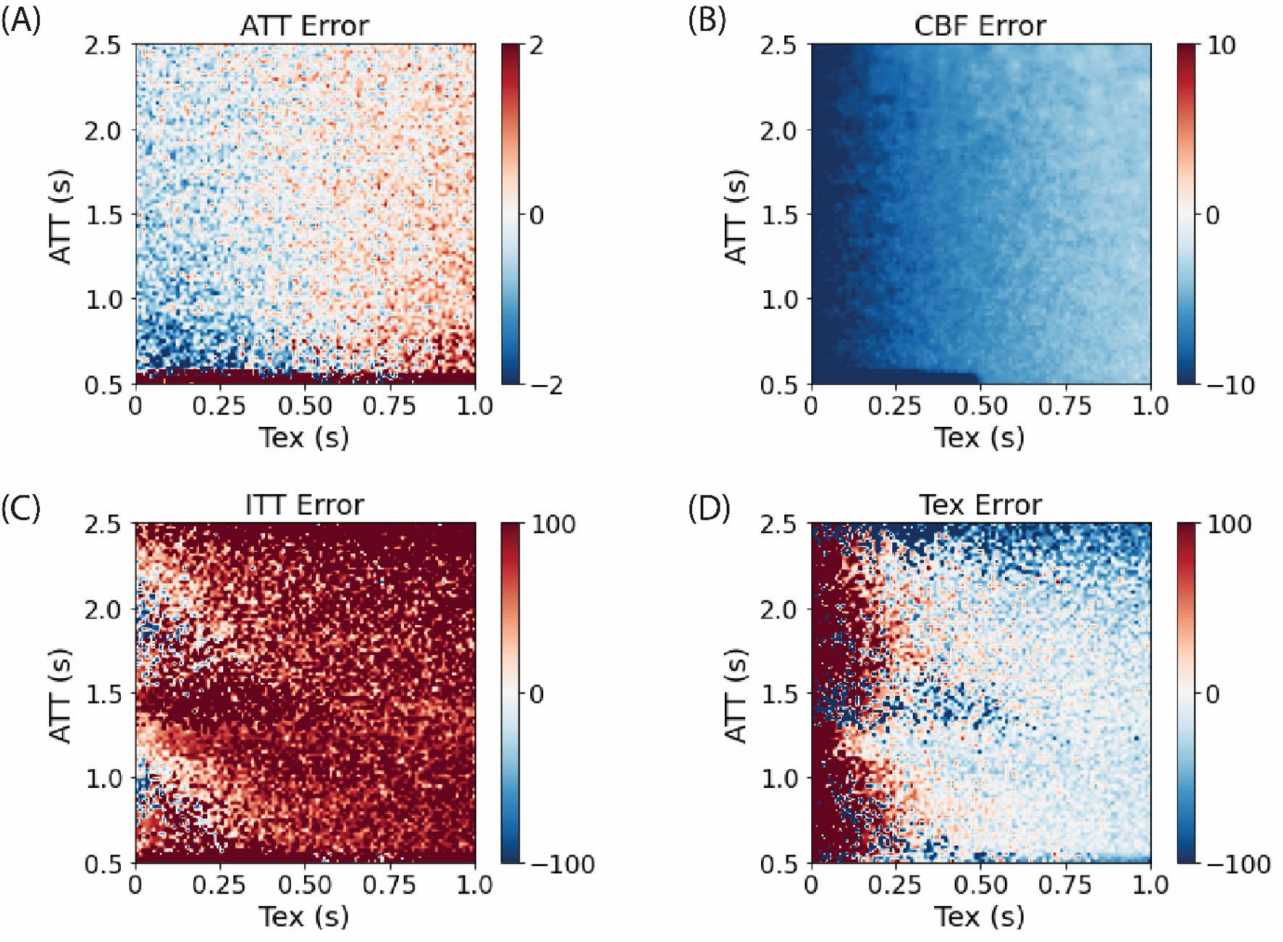
Error maps for estimated parameters across a range of simulated ATT and Tex values using the two-stage approach. Each panel displays the error in a specific parameter: **A** ATT error, **B** CBF error, **C** ITT error, and **D** Tex error

To compare the accuracy of the two approaches, relative errors against the ground truth data were calculated. Figures 2 and 3 show errors in fitted parameters resulting from one-stage approach and the two-stage approach, respectively. The one-stage estimation yielded robust results across all four parameters, with minimal interdependence between Tex and ITT. However, slight cross-talk was observed at lower Tex values (0–100 ms). In contrast, the two-stage approach showed a 2% error in ATT and up to 10% underestimation of CBF in the initial stage. This underestimation of CBF propagated into the second stage, causing a significant overestimation of ITT, while Tex estimation remained relatively stable, with errors at low values which seem to appear as interdependence with ITT.

The simulations suggest that the two-stage approach, which applies different models at each stage, is vulnerable to error propagation due to parameter cross-dependence. Specifically, the Buxton model led to up to a 10% underestimation of CBF. This underestimation can be attributed to the limitations of single-TE data and the single-TE-based Buxton model, which relies on a single time point to estimate signal decay. This approach may assume a slower decay rate because of only one TE time point, potentially leading to a lower intercept and, consequently, a lower CBF estimate. In contrast, multi-TE data and models that incorporate multiple TEs better capture the true signal decay, which may be faster in reality. This more accurate characterization of decay likely results in a higher intercept and explains the higher CBF estimates observed with the multi-TE approach.

These fixed estimates of CBF and ATT in the second step propagated errors into ITT, resulting in considerable overestimation, while Tex remained relatively stable as it is primarily dependent on T2 changes in tissue and blood. Moreover, these results highlight that the physiological fluctuations and errors tend to affect ITT, leaving Tex estimation stable, highlighting the importance of separating the phenomena of transit within the voxel and exchange time. Both approaches showed some interdependence between Tex and ITT at low Tex values, suggesting that additional signal weighting (such as diffusion weighting) is required to accurately separate Tex and ITT and minimize cross-talk. Nevertheless, with expected Tex values in the 200–500 ms range, both approaches produced relatively robust estimates for Tex with minimum error.

Both modeling approaches yielded stable Tex estimates; however, the one-stage approach minimized cross-dependence issues and provided more reliable CBF and ITT accuracy. Hence it was adopted for the in vivo data analysis.

### Imaging

Ten healthy volunteers (age 31 ± 9 years, 3 females) were examined at 3 T (MAGNETOM Vida Fit, Siemens Healthineers AG) using a 20-channel head coil. A written informed consent was provided by all volunteers before scanning. The study was conducted under a general protocol for pulse-sequence development approved by the ethical committee of the University of Bremen, Bremen, Germany. All volunteers were regular coffee drinkers, reporting an average consumption of two cups of coffee per day. Every volunteer was scanned in the morning in a fasting state and was instructed to avoid caffeine intake for at least 8 h prior to the scan.

**Fig. 4:**
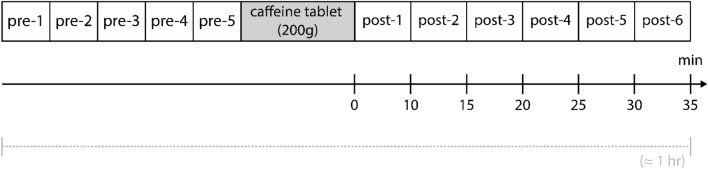
Overview of study design. A BBB-ASL protocol was designed using a combination of single-TE and multi-TE Hadamard measurements aimed at estimating exchange time as a proxy measure of blood–brain barrier permeability. Five measurements were acquired as a baseline to observe fluctuations in physiological parameters before administering caffeine, represented here as ‘pre 1–5’. Six measurements were taken after administering a caffeine tablet (200 mg) to the volunteers, shown here as ‘post 1–6’

Five sets of baseline pre-caffeine ASL and M0 scans, each 04:50 min long, were acquired to evaluate fluctuations in physiological parameters. After acquiring the baseline sets, the volunteers were taken out of the scanner and given a 200 mg caffeine tablet while remaining in the supine position, then immediately placed back into the scanner without any delay. Six sets of post-caffeine ASL and M0 scans were acquired, covering the post-caffeine dynamics for approximately 35 min. Figure 4 provides a visual representation of the study design.

A combination of single-TE and multi-TE Hadamard pseudo-continuous arterial spin labeling (pCASL) sequence [22], implemented in the in-house developed vendor-independent MRI framework gammaSTAR [23, 24] with 3D GRASE readout [25] was used. Two measurements of multi-TI, single-TE data were acquired using Hadamard-8 (HAD-8) matrix with a sub-bolus duration (SBD) of 400 ms and a post-labeling delay (PLD) of 600 ms and 800 ms, respectively (TE = 13.2 ms, TR = 4000 ms, turbo factor = 12, and scan time = 02:15 min). The resulting two sets of seven inflow times (TI, where TI = SBD + PLD) ranged from 1000 to 3400 ms with an increment of 400 ms, and from 1200 to 3600 ms with an increment of 400 ms, respectively. A multi-TI, multi-TE data was acquired using Hadamard-4 (HAD-4) matrix with SBD of 1000 ms and PLD of 500 ms. The resulting three TIs were 1500 ms, 2500 ms and 3500 ms (TR = 4500 ms, turbo factor = 2, and scan time: 01:55 min) and each TI was acquired at eight different echo times ranging from 13.8 ms to 207 ms with an increment of 27.6 ms. Two FOCI pulses were used for background suppression of T1 values 700 ms and 1400 ms. All pCASL measurements were acquired with the in-plane field of view (FOV) = 320×160 mm^2^, matrix size = 64×32x32, nominal spatial resolution = 5×5x5 mm^3^, EPI factor = 16, bandwidth = 2300 Hz/Px, slice partial Fourier = 6/8, one pre-scan and an acceleration of 2 × 2 with CAIPIRINHA. M0 images were acquired in RL and LR phase encoding directions for distortion correction and to quantify perfusion (TE = 13.2 ms, TR = 5000 ms, TIs = 300, 1300, 2300 ms, scan time = 00:20 min). A T1 MPRAGE was acquired with the following parameters: TR = 2200 ms, TE = 2.98 ms, inversion time (TI) = 900 ms, flip angle = 9°, FOV = 256 mm^2^, voxel size = 1×1x1 mm^3^, matrix size = 224×256x256, sagittal orientation, and scan duration = 05:07 min.

### Data analysis

Data were analyzed with an in-house developed pipeline using Oxford Centre for Functional MRI of the Brain (FMRIB)’s Software Library (FSL) [21]. Structural T1 MPRAGE images were preprocessed with fsl_anat. The ASL time series were corrected for motion using MCFLIRT, employing a six-parameter rigid transformation, and distortion corrected using M0 images acquired in phase-reversed directions (RL and LR) with the FSL TOPUP module [26]. ASL signal at each TI and TE was decoded by applying the respective Hadamard decoding matrix.

All ASL data were concatenated and fitted to estimate CBF, ATT, Tex, and ITT using the extended two-compartment multi-TE model incorporated into the Bayesian non-linear fitting framework of FSL FABBER [20]. Mean gray matter values were calculated using a 50% probability gray matter mask. The parameter maps were registered to structural and MNI 152 standard spaces to compare them within and across subjects.

A mixed-effect model was applied to investigate the time-dependent change in estimated parameters using R (RStudio 2024.04.2 + 764). Additionally, pre-caffeine measurements were averaged across subjects to create a baseline, which was compared with the last post-caffeine measurement using a two-tailed paired Student’s *t*-test.

## Results

**Fig. 5:**
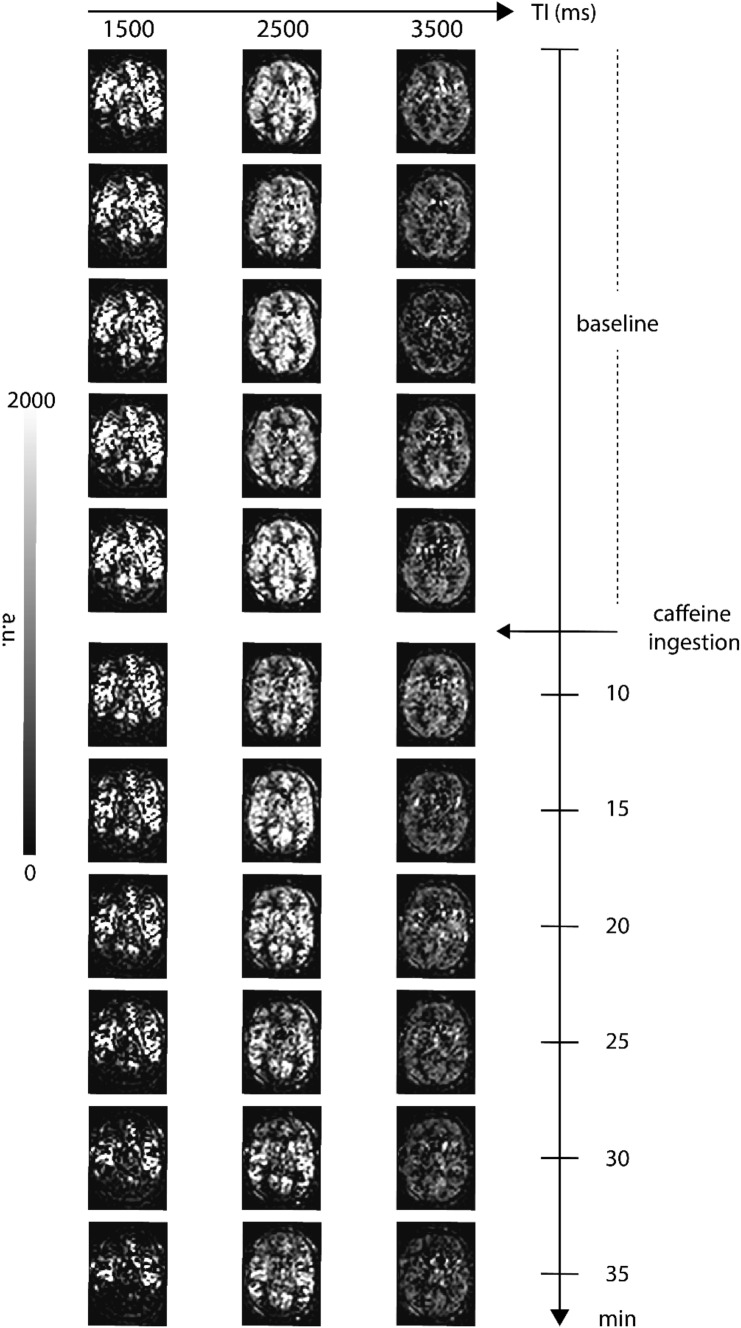
HAD-4 ASL (TE-1) decoded images from a representative volunteer showing pre-caffeine and post-caffeine sets, comparing changes in signal over time following caffeine ingestion

**Fig. 6:**
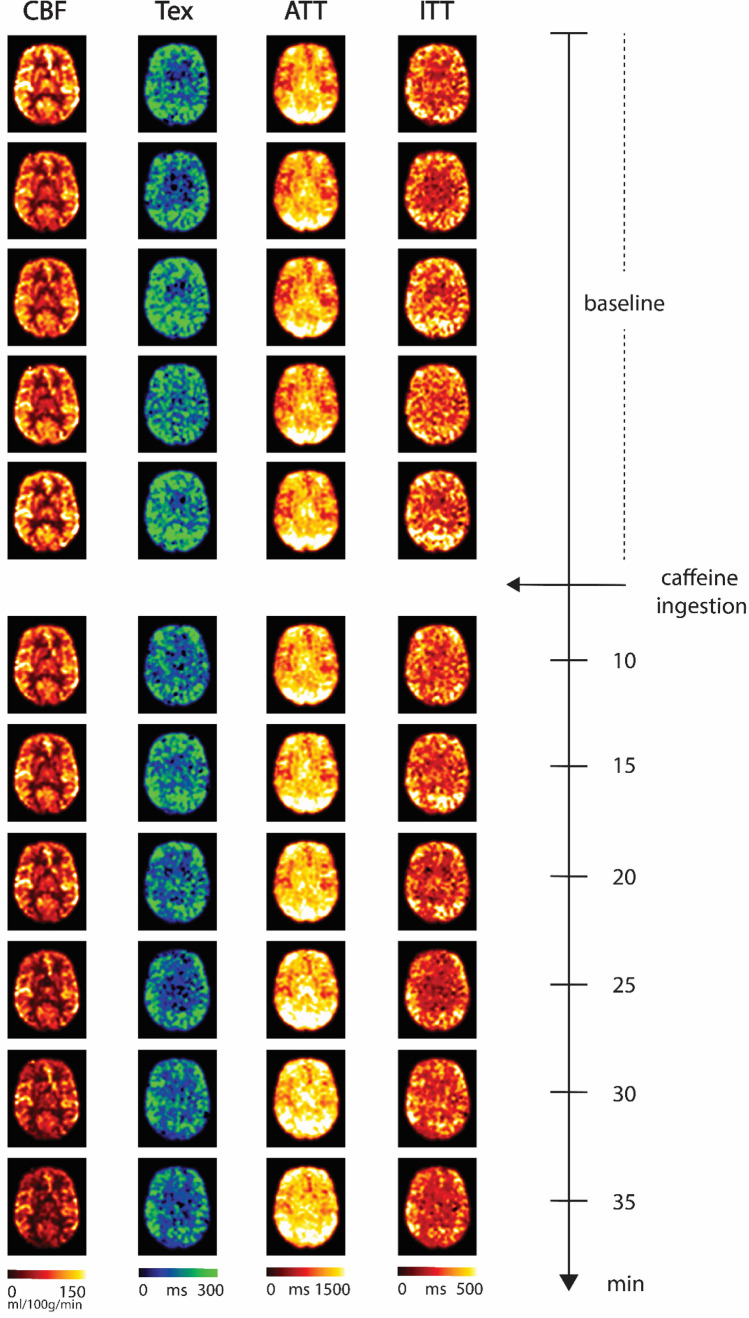
Fitted parameter maps of cerebral blood flow (CBF), exchange time (Tex), arterial transit time (ATT), and intra-voxel transit time (ITT) for the same representative volunteer

**Fig. 7:**
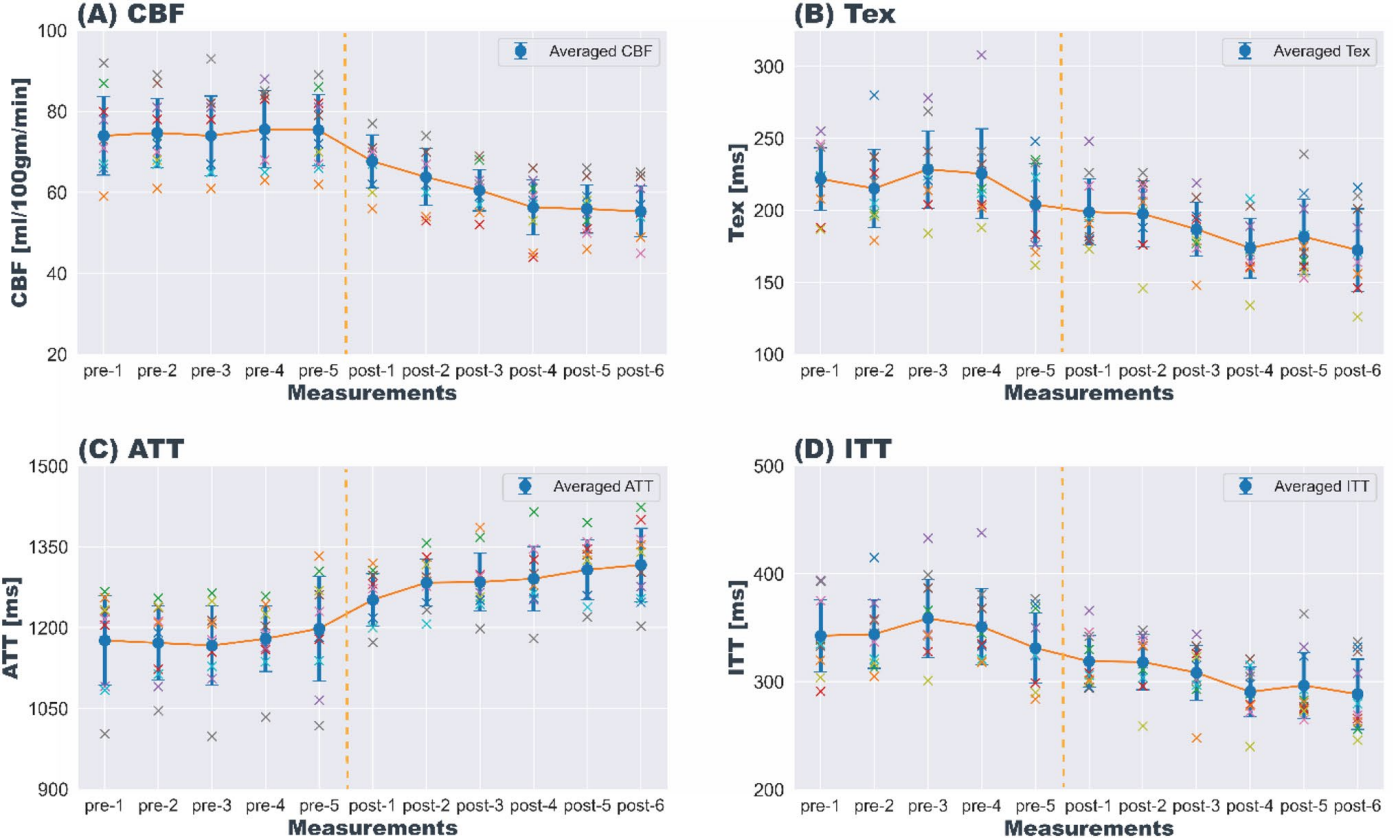
Changes in the dynamics of physiological parameters **A** CBF, **B** Tex, **C** ATT, and **D** ITT pre- and post-caffeine ingestion. Five pre-caffeine measurements were acquired as a baseline. The vertical dotted orange line shows the time of caffeine ingestion. Six post-caffeine ingestion measurements were acquired, covering a total of approximately 35 min after caffeine intake. Each ASL measurement was 4:50 min long. Error bars show the standard deviation across volunteers

Figure 5 shows three TIs of decoded HAD-4 (TE-1) images from pre-caffeine sets and six post-caffeine sets for a representative subject. After caffeine intake, all three TIs showed a decrease in signal over time. Figure 6 shows fitted parameter maps, including quantified CBF, Tex, ATT, and ITT for the same subject. Plots representing a dynamic change in the fitted parameters during pre-caffeine and post-caffeine sets are shown in Fig. 7. As can be seen, after caffeine intake, CBF, Tex, and ITT prominently decreased while ATT increased. Table 1 shows the mean gray matter parameter values from all pre- and post-caffeine sets, averaged across all subjects.

**Table 1:** Summary of mean gray matter values of fitted parameters averaged across all volunteers

set	CBF [ml/100gm/min]	Tex [ms]	ATT [ms]	ITT [ms]
pre-1	74 ± 10	222 ± 23	1176 ± 87	343 ± 35
pre-2	75 ± 9	215 ± 29	1172 ± 72	344 ± 34
pre-3	74 ± 10	229 ± 28	1167 ± 77	359 ± 38
pre-4	76 ± 10	226 ± 33	1179 ± 64	351 ± 37
pre-5	75 ± 9	204 ± 30	1198 ± 102	331 ± 34
post-1	68 ± 7	199 ± 24	1252 ± 51	319 ± 25
post-2	64 ± 7	198 ± 24	1284 ± 46	318 ± 27
post-3	61 ± 6	187 ± 20	1284 ± 57	308 ± 27
post-4	56 ± 7	174 ± 22	1291 ± 63	291 ± 24
post-5	56 ± 6	182 ± 28	1307 ± 58	296 ± 32
post-6	55 ± 7	172 ± 30	1316 ± 71	288 ± 34

Table 2 shows the results of the mixed-effects model which revealed that following caffeine ingestion, CBF decreased significantly (*P* < 0.01) over time while Tex and ITT showed a non-significant decrease, (*P* = 0.234 and *P* = 0.0674, respectively). Conversely, ATT showed a non-significant increase (*P* = 0.0984).

**Table 2:** Summary of mixed effects model for physiological parameters

Parameter	Estimate	Standard Error	*p*-value	conf. low 2.5%	conf. high 97.5%
CBF	– 0.5889	0.1036	< 0.001	– 0.7919	– 0.386
Tex	– 0.602	0.506	0.234	– 1.59	0.391
ATT	1.25	0.754	0.0984	– 0.232	2.72
ITT	– 1.04	0.568	0.0674	– 2.15	0.0743

**Fig. 8:**
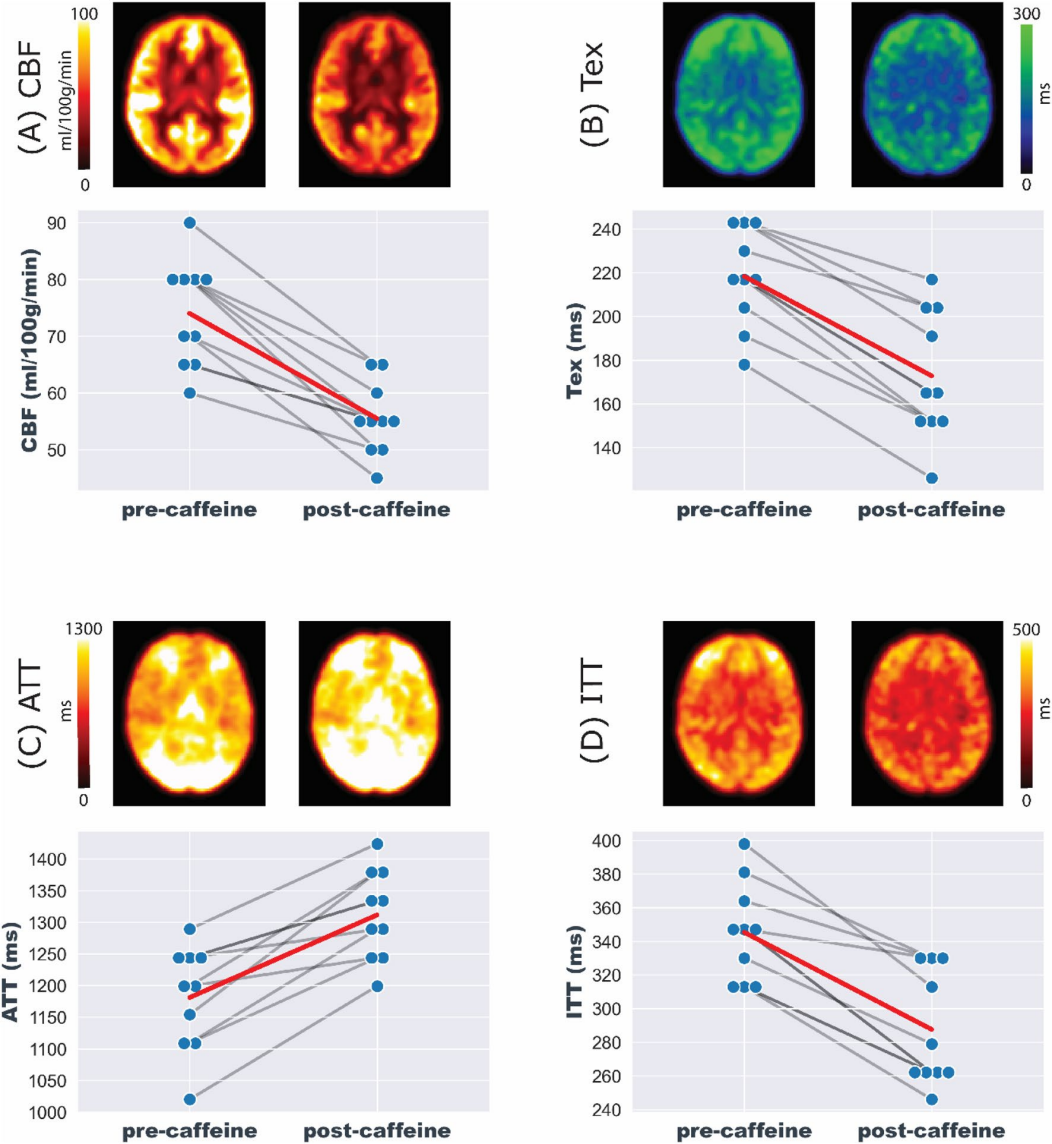
Comparison of parameter maps averaged across all volunteers for **A** CBF, **B** Tex, **C** ATT, and **D** ITT from pre-caffeine (averaged) and post-caffeine (post-6) measurements. The ladder plots illustrate the magnitude and direction of changes in these parameters following caffeine ingestion, highlighting the overall trend. An average change is shown in red, along with the individual responses of the volunteers

Comparing the averaged baseline measurements with the last post-caffeine measurement revealed a 26% decrease in CBF (*P* < 0.01), a 21% decrease in Tex (*P* < 0.01), a 17% decrease in ITT (*P* < 0.01), and a 12% increase in ATT (*P* < 0.01), as shown in Fig. 8.

## Discussion

In this study, the impact of caffeine ingestion on BBB permeability to water using multi-TI, multi-TE ASL imaging was evaluated. These results showed that after caffeine ingestion, the apparent exchange time between the microvascular and the tissue compartments did not show statistically significant time-dependent change, although a non-significant decreasing trend in Tex estimation was observed. Under the assumptions of the underlying model, this reflects faster movement of labeled water across the BBB. Furthermore, it was found that CBF reduced significantly, which is in line with previous studies [6, 7, 27].

While the time-dependent changes in BBB permeability were not statistically significant, we observed a significant difference when comparing the averaged baseline measurement with the last post-caffeine measurement, suggesting an overall trend of increased permeability following caffeine ingestion. A larger dataset would likely be required to achieve statistical significance in time-dependent changes and to account for potential variability between subjects. It is also possible that individual differences in caffeine metabolism and habitual caffeine intake could influence the extent of BBB permeability changes, as these factors might affect how each subject physiologically responds to caffeine ingestion. Future studies with larger sample sizes and consideration of individual caffeine intake history could provide more robust insights into the observed trends and their implications.

The role of the BBB in preserving homeostasis within the delicate brain tissue has been extensively reported, and interest in developing noninvasive imaging techniques has recently increased to assess this neuroprotective function of the BBB in both healthy and pathological states. This study aimed to assess changes in water exchange dynamics, reflecting BBB function, under a perturbed physiological condition in healthy humans. For this purpose, caffeine, a known psychostimulant and adenosine antagonist that modulates brain hemodynamics by influencing CBF, was used. While prior studies have highlighted the neuroprotective role of caffeine in various pathologies, including Parkinson’s disease, Alzheimer’s disease, and in rat models of chronic sleep restriction [4, 28], these studies were largely conducted in disease models where the BBB was already compromised. Such studies primarily focus on chronic caffeine intake, which may upregulate adenosine receptors [1] and influence BBB permeability over time. In contrast, the current study differs in its focus on evaluating the acute effects of caffeine ingestion on BBB water permeability in healthy humans with an intact BBB. Using the multi-TE ASL approach, we aimed to investigate transient physiological changes induced by caffeine under controlled conditions. Unlike the chronic effects studied in prior research, our results reflect short-term changes in BBB permeability dynamics, providing a baseline for understanding acute caffeine effects on BBB function in healthy states. Future research could further investigate these effects in neurodegenerative disease populations or with chronic caffeine exposure.

The role of caffeine in vasoconstriction has been validated by various techniques including transcranial Doppler, PET, and MRI [6, 7, 29]. The reduced CBF found in this study is consistent with the 15–25% decrease reported in previous caffeine challenge studies [3]. Adenosine, being an inhibitory neurotransmitter, decreases the release of synaptic vesicles in the presynaptic terminal. Therefore, considering that caffeine acts as an antagonist to adenosine, it would be reasonable to assume that caffeine ingestion would result in increased neural activity and whole-brain metabolic rate (CMRO2). Conversely, previous studies found that CMRO2 remained constant while the oxygen extraction fraction (OEF) increased significantly as compensation in response to decreased CBF [3, 30]. Similar to this, our finding of increased water exchange could be a compensatory mechanism to maintain brain homeostasis in response to caffeine-induced reduction in CBF.

Recently, a study using the water-extraction-with-phase-contrast-arterial-spin-tagging (WEPCAST) technique, where the ASL signal is selectively measured in the draining veins of the brain, reported an increased water extraction fraction (E) in brain tissue in response to a caffeine challenge [16]. Along with E, the authors reported that the permeability surface area product (PS) remained unchanged. In the present study, exchange time was explicitly measured, which reflects the transition of labeled spins from blood into the tissue, based on the change in transverse T2 relaxation. Our method is sensitive only to this blood-to-tissue transition and does not consider the surface area of the vessels. Moreover, considering our findings that water flux increases, combined with the assumption that the vessel surface area decreases due to vasoconstriction, our results are consistent with the unchanged PS value reported in the WEPCAST study. On the other hand, a study using a diffusion-based method called Intrinsic Diffusivity Encoding of Arterial Labeled Spin (IDEALS) investigated the caffeine-induced BBB response in four subjects and found that both the water extraction fraction and PS decreased after caffeine intake [27]. One explanation for these contrasting results compared to our study could be that the two approaches probe different properties of labeled blood water: transverse relaxation and diffusivity. These properties might change significantly at different times and locations, capturing different stages or time courses of the water exchange mechanism. If one property changes earlier than the other, it could provide different results, as we are witnessing. To effectively capture these changes, it would be necessary to design ASL protocols and sampling times tailored to each technique. Further research is needed to compare water-based MRI methods to fully understand the origin of the BBB signal being measured.

In addition to decreased CBF in response to caffeine ingestion, we also observed trends of prolonged ATT and reduced ITT. It has been argued in previous studies that caffeine, eliciting numerous complex mechanisms of action, could have dual effects on the vascular system [31]. It may act as a vasodilator in the cardiovascular system but cause vasoconstriction in cerebral arteries [31, 32]. The physiological changes governing cerebral flow velocity could be explained by the myogenic hypothesis [33], which states that the diameter of small perfusion vessels could modulate in response to cognitive or functional requirements. Such changes in the lumen of the vessel could alter cerebral blood supply and, consequently, change blood velocity. This may involve only smaller vessels, like arterioles, as the diameter of the middle cerebral artery has been reported to remain unchanged during numerous autonomic neural challenges [34, 35]. The opposing trends of change in transit times of ATT and ITT may be caused by such dual effects of caffeine on the vascular system.

Reduced ITT in response to caffeine intake could be interpreted as increased blood velocity through smaller arteries and arterioles, due to vasoconstriction, resulting in shorter transit time. Moreover, studies have shown that CBF modulation may result in capillary transit time heterogeneity (CTTH) to compensate for brain tissue oxygenation [36]. A study investigating cerebral microcirculation in Alzheimer’s disease found that decreased CBF was associated with disturbed capillary flow patterns, which might serve to maintain efficient oxygen extraction during a perturbed perfusion state as a compensatory mechanism [30]. Our observation of reduced ITT, indicating increased blood velocity, may be explained by similar capillary flow disturbances in response to decreased CBF, though further studies are needed to validate such a phenomenon. A second explanation could be that the exchange process starts earlier while the labeled blood is still traversing through the arterioles within a voxel. Although it is considered that water exchange only takes place at the capillary bed, terminating arterioles and post-capillary venules are also reported to be surrounded by astrocytic end-feet carrying aquaporins, enabling these parts of the vessels to participate in water exchange to a limited extent [37]. A compensatory mechanism of increased water flux in these vessel segments may have occurred in response to decreased perfusion to maintain water homeostasis before the labeled water reached the capillary bed, thus resulting in reduced ITT.

The current study used caffeine tablets to ensure a controlled and reproducible investigation of caffeine’s acute effects on brain physiology. This approach isolates caffeine’s role as an adenosine receptor antagonist without the confounding influence of other bioactive compounds present in coffee or additives in energy drinks. Coffee, for example, contains over 1000 bioactive compounds, such as chlorogenic acids and polyphenols, which are known to have antioxidant and anti-inflammatory effects and may synergize with caffeine to enhance its neuroprotective properties [38]. Additionally, the lipid content in coffee may alter caffeine absorption, potentially modulating its impact on brain perfusion [39]. In contrast, energy drinks often include high sugar content and other additives, which could affect vascular responses and complicate the interpretation of caffeine’s direct effects [40]. While the tablet form offers significant advantages for studying caffeine's specific effects, future studies could explore how the consumption of coffee or energy drinks influences brain perfusion and BBB permeability in comparison to pure caffeine. Such investigations would provide insights into whether the neuroprotective effects attributed to caffeine are enhanced or modulated by other compounds present in regular coffee and other beverages.

The current study has a few limitations. First, we measured approximately 35 min of post-caffeine dynamics, which is within the range of reported times for caffeine to reach a maximum plasmatic concentration in a fasting state [38, 41]. However, covering additional time would provide a better understanding of the relative steady state, where a plateau would be expected, followed by the restoration of all physiological parameters. Second, we scanned regular coffee drinkers but used a fixed amount of caffeine for the challenge (200 mg). This could have resulted in ‘between-subject variability,’ as caffeine is known to have a dose-dependent effect, where chronic intake of caffeine may lead to the upregulation of adenosine receptors [1]. Moreover, withdrawal effects of caffeine have been reported in moderate and high coffee users, which may influence physiological parameters; for example, CBF is reported to increase when caffeine is abstained from by a regular coffee drinker [6]. Lastly, we did not assess the regional heterogeneity of caffeine-induced decreases in CBF as reported by previous studies [3]. Such an analysis could provide information about the spatial distribution of adenosine receptors and possible heterogeneity in water exchange dynamics in the brain.

## Conclusion

In conclusion, this study evaluated the effect of caffeine ingestion on BBB water permeability by measuring the exchange time of labeled water across the BBB. The results suggest that water permeability increased in response to caffeine intake, which could be a compensatory mechanism to counteract decreased CBF and maintain homeostasis in the brain. The study provides evidence that the non-invasive multi-TE ASL method can detect physiological changes occurring in the healthy human brain, offering encouragement to further explore pathological conditions to better understand the underlying physiological interactions in the brain.

## Data Availability

Participants of this study did not agree for their data to be shared publicly.
